# Cold applications for recovery in adolescent athletes: a systematic review and meta analysis

**DOI:** 10.1186/s13728-015-0035-8

**Published:** 2015-10-12

**Authors:** Andrew Murray, Marco Cardinale

**Affiliations:** Department of Sports Science, Aspire Academy, Doha, Qatar; University of Edinburgh, Edinburgh, UK; Department of Computer Science and Institute of Sport Exercise and Health, University College London, London, UK

**Keywords:** Recovery, Ice, Cold, Youth, Adolescent, Athlete, Elite

## Abstract

Recovery and regeneration modalities have been developed empirically over the years to help and support training programmes aimed at maximizing athletic performance. Professional athletes undergo numerous training sessions, characterized by differing modalities of varying volumes and intensities, with the aim of physiological adaptation leading to improved performance. Scientific support to athletes focuses on improving the chances of a training programme producing the largest adaptive response. In competition it is mainly targeted at maximizing the chances of optimal performance and recovery when high performance levels are required repeatedly in quick succession (e.g. heats/finals). In recent years, a lot of emphasis has been put on recovery modalities. In particular, emphasis has been placed on the need to reduce the delayed onset of muscle soreness (DOMS) typically evident following training and competitive activities inducing a certain degree of muscle damage. One of the most used recovery modalities consists of cold-water immersion and/or ice/cold applications to muscles affected by DOMS. While the scientific literature has provided a rationale for such modalities to reduce pain in athletes and recreationally active adults, it is doubtful if this rationale is appropriate to aid training with adolescent athletes. In particular, since these methods have been suggested to potentially impair the muscle remodeling process leading to muscle hypertrophy. While this debate is still active in the literature, many coaches adopt such practices in youth populations, simply transferring what they see in elite sportspeople directly; without questioning the rationale, safety or effectiveness as well as the potential for such activity to reduce the adaptive potential of skeletal muscle remodeling in adolescent athletes. The aim of this review was to assess the current knowledge base on the use of ice/cold applications for recovery purposes in adolescent athletes in order to provide useful guidelines for sports scientists, medical practitioners, physiotherapists and coaches working with such populations as well as developing research questions for further research activities in this area. Based on the current evidence, it seems clear that evidence for acute benefits of such interventions are scarce and more work is needed to ascertain the physiological implications on a pre or peri-pubertal population.

**Electronic supplementary material:**

The online version of this article (doi:10.1186/s13728-015-0035-8) contains supplementary material, which is available to authorized users.

## Background

The typical structure of any training programme aimed at enhancing human performance is based around the principle of overload. The most common example of a training programme consists of cycles of progressively intensified training separated by a drop in volume, intensity or both or recovery weeks across a season; this cyclical approach has been suggested to disrupt the physiologic homeostasis through training so that the performance capacity improves via supercompensation [1, 2]. To maximize the effectiveness of such a programme, there is the need to subject the athlete to not only periods of intensive training loads but crucially the appropriate recovery to facilitate adaptation [3].

Such an approach is used by coaches worldwide not only to train adult athletes, but also to develop athletic potential in adolescents^1^. Unlike adults, the evidence for overtraining in young athletes does not seem to be strong, probably limited by the lack of appropriate longitudinal studies [4]. Muscle damage is one of the many well-known physiological effects of increased loading, in particular when resistance exercise and/or repeated eccentric actions are employed [5–9]. When considering training planning, the extent of the ‘damage’ can be placed on a continuum from excessive (injury) to the minimum dose needed to cause a training effect. In recent years, a lot of studies and empirical activities have been targeted to identify the best recovery strategies for the athlete and coach to reduce any negative implications of training loads to allow quicker recovery whilst maximizing adaptation. Despite the fact that recovery is recognized as a need when planning the training activities of an athlete, a paucity of data exists on the implications of various recovery activities. There is no definition of the most ‘appropriate’ modality, protocol and timing according to the level of the athlete and their training goals [10].

Recent work has highlighted that the aim of most recovery interventions is to return psychological [11], physiological [12, 13], and performance variables [14] to the ‘pre-exercise’ level or to baseline conditions identified in the absence of fatigue. There have been a number of reviews in recent years on the use of recovery modalities within elite adult sport. The main focus for these has been on therapies utilizing different temperature mediums; cold in the form of water immersion and whole body cryotherapy [15–18] and heat via saunas and water immersion [19, 20]. A recent meta-analysis has suggested a large effect of cooling after exercise on the ability to retain sprint performance but much lower effects on endurance or strength [16]. In all cases, performances were most affected at 4 days following the exercise session/insult used to study the effectiveness of the recovery intervention. On average though, these effects seemed relatively small (Hedges effect size = 0.28). However, a more recent review still supported the idea that such interventions can useful enough for competitive athletes, provided ‘appropriate’ methodologies can be utilized [19]. Appropriateness should refer to the aim and the expected outcome of an intervention. Despite the fact that cold-water immersion (CWI) seems to be an effective (and therefore appropriate) strategy to reduce delayed onset muscle soreness (DOMS), it does not seem effective in restoring strength and might have some potential to restore power [21] in adult athletes. However, while some justification seems to be warranted for the use of such modalities in elite athletes, there is a paucity of data on the rationale and effectiveness for younger ones be they elite or amateur.

As muscle damage is one of the well-known physiological effects of increased loading, exercise protocols capable of creating muscle damage have mostly been used as the model to understand the effectiveness of recovery modalities. The main rationale being the ability of such a stimulus to induce marked changes in the skeletal muscles of individuals unaccustomed to resistance and/or eccentric exercise; from alterations at the cellular level to observed reductions in indices of muscle function [22]. The discomfort and soreness associated with muscle damage generally increases and peaks between 24 and 72 h following the exercise/training bout causing it, before subsiding (if there is no further training) over the next 5–7 days [23] as the injury is gradually repaired [24, 25]. When the training is repeated, a marked reduction in muscle soreness and an increase in inflammatory markers is observed and this phenomenon has been termed the repeated bout effect, indicating an adaptive response to the training stimulus [26–28]. It is nowadays accepted that while the exact mechanism is not yet elucidated [29] the trained or preconditioned individual [30] should not be prone to the same levels of muscle damage and associated symptoms typically observed in the untrained if they use similar exercises in the training regime [31]. The objective of any inflammatory process, is to repair injury and restore tissue function [32], (for a review of the mechanisms involved in muscle damage see [33]). This is one of the reasons why coaches prescribe varied forms of exercise, were the intensity and volume of the activities and the modalities of contractile activity are such that they create micro-trauma to foster the remodeling of muscle tissue. This training approach is aimed at triggering an adaptive response such that the muscle is more resistant to damage and any damage that does occur is repaired at a faster rate. It is also clear that a relatively small insult will produce this adaptation [34–36]. However, as inflammation and pain reduce the ability to train or compete, various approaches are taken to reduce the symptoms.

In an attempt to reduce the negative symptoms of inflammation, recovery techniques have been mainly targeted to affect skeletal muscles’ temperature and blood flow [17, 37–44]. The most popular approach involves CWI, which combines the effects of cold and increase in hydrostatic pressure to trigger multiple physiological effects capable of reducing pain perception and facilitate recovery. Research on young athletes is somewhat limited with regards to both the adaptation to training and the effects of various recovery modalities due to methodological and ethical constraints. The limited evidence available advocates that pre-pubertal youths recover quicker than adults [45] mainly due to their lower relative power capabilities, but also due to a relative larger flexibility and muscle compliance which makes them less susceptible to muscle damage than their adult counterparts [46–49]. Recent work [50] has shown that muscle damage (and its symptoms) is a lot less in pre-adolescents than adolescents and adults when similar eccentric exercise protocols and loading patterns were applied. Furthermore, the few previous studies published in pre and post-pubertal populations have clearly presented that the extent of muscle damage in children is less than adults [51, 52].

Despite the scarcity of research studies in this field, coaches and parents sometimes train and recover adolescent athletes in the same way as they would with adult populations, mostly attracted by the popular headlines of lay press and/or replicating what they see in professional sport. Considering the lack of scientific consensus on this topic and the need to develop an evidence-based approach to recovery strategies in adolescent athletes, we aimed to review the current literature with a specific focus on cold applications to identify safe and effective interventions currently known, explore the potential adaptive mechanisms and suggest new avenues for research in this field.

## The rationale for cold applications and training adaptations in adolescent athletes

### Exercise, inflammation and muscle remodeling

After high intensity exercise there is typically an acute performance reduction due to mechanical and/or metabolic stresses [21, 53, 54]. Exercise that induces a large metabolic stress (such as endurance or interval training) contributes to an increase in reactive oxygen species generation (ROS; [24, 55]). ROS have been shown to determine many acute and chronic alterations in skeletal muscle mainly characterized by marked inflammatory responses (for a review see [56]).

While there are a number of papers in the literature involving adult athletes, limited information exist on adolescent athletes. Recent work proposed that the resting blood redox balance is well maintained in adolescent endurance athletes due to adaptation to exercise [57]. Furthermore, Kabasakalis and colleagues suggested that children, involved in intensive swimming training, could improve their antioxidant capacity in the same manner as adult athletes [58]. As previously stated, when damaging exercise activities are performed, the initial insult causes a mechanical disruption of the muscle fibers. Subsequent damage is linked to inflammatory processes over the next 24 h and to changes in excitation–contraction coupling within the muscle [33]. The inflammation sees a proliferation of fluid, plasma proteins and inflammatory cells [59–61] which is thought to amplify the initial muscle injury through increased ROS release and activation of phospholipases and proteases at the injury site [59].

Within an adult population, the time course of neutrophil accumulation (the primary step in the inflammation cascade) varies from as little as 45 min after eccentric exercise [62] up to 5 h [59], dependent on both the type and intensity of exercise [63]. The subsequent course of the inflammation process depends on the relative balance between increases in pro-inflammatory (interleukin [IL]-1, IL-6, tumor necrosis factor) and anti-inflammatory cytokines (IL-10), to produce a sufficient inflammatory response to adequately repair injured tissue without creating an excessive response [64, 65].

The majority of published studies in adult populations have reported increased concentrations of IL-6 in the blood after both prolonged endurance and eccentric exercise [65–69], suggesting its usefulness as a biomarker to understand inflammatory responses to exercise. Further work has also hypothesised a relationship of IL-6 with intensity and duration of exercise [70]. In fact, it has been shown that the release in the bloodstream is higher after prolonged endurance rather than brief maximal exercise. After 12 h of exercise, intensity seems to be the key determinant whereas under 12 h, it is duration [70]. Furthermore, training status may also affect the IL-6 response with well-trained runners presenting lower IL-6 concentrations than untrained ones [71, 72]. Yet there are conflicting reports in the literature as higher post exercise responses in IL-6 were also positively related to performance improvements in highly trained male rowers [73]. It is almost impossible to define the timeline of IL-6 kinetics in the bloodstream due to the varied protocols and timelines in the literature. Despite the surge in interest in interleukins and intense/damaging exercise the response in adolescent athletic populations is somewhat limited. Volleyball training in elite adolescent athletes has been shown to determine marked inflammatory responses [74] characterized by marked increases in IL-6. Cycling exercise for 60 min at an intensity of 70 % of VO2 max was also shown to determine marked increases in leukocytes, lymphocytes and cytokines in healthy boys and girls aged 12–14 [75]. Tennis practice was also shown to determine marked inflammatory responses in healthy adolescents regularly engaged in tennis training (more than 17 h per week) [76]. Finally, acute bouts of wrestling [77] performed by high school athletes were capable of causing an acute increase in pro-inflammatory cytokines after the sessions, as well as chronically over the course of the high school sporting season. This suggests that increased training and competition loads in contact sports can trigger significant inflammatory responses in young athletes [78]. Limited studies exist on the consequences of eccentric resistance exercise in pre-, peri- and post-pubertal athletes.

## Adolescents vs adults: differences in inflammation and muscle remodeling

Adolescent boys, similarly to adults, demonstrate enzymatic muscle changes specific to the modality of training employed similar to adults [79], albeit with lower magnitudes [80, 81]. This has been proposed as an explanation why recovery can be quicker than that of their adult counterparts [52]. Despite the fact that adolescent athletes show similar adaptations to exercise as adults, they seem to not experience similar symptoms of muscle damage after intense exercise [46, 50, 51, 82]. Adequate training loading is necessary in young populations as excessive training volumes and intensities and inappropriate progressions in a young athlete can create overload and injury whilst the correct amount can promote growth and performance gains [83, 84].

Serum creatine kinase (CK) has been proposed as an indirect marker of muscle damage mainly because it can be determined with inexpensive assays and point of care testing. Although caution needs to be used as it is highly individual in its variability, its concentration in blood is affected by the training level and the muscle groups involved in exercise, to a greater extent than differences in exercise volume or intensity [85]. In the sporting domain, most decisions regarding recovery interventions following intense training sessions are based on CK levels, however some caution needs to be applied when considering the implications for young athletes. In fact, the observed circulating levels of CK are typically lower in the young and elderly following exercise of the same relative intensity when compared to adults. Using CK as the unique marker to determine the need for an intervention to reduce inflammation may not represent the best informed decision in an adolescent group. In fact, Webber and colleagues investigated the CK response following a bout of downhill running, and observed a significantly greater CK increase in adults in comparison with prepubescent children [86]. However, when body weight was accounted for, no significant age effects were observed, leading the authors to conclude that different increases in CK between adults and children were related to body weight. Other authors have reported that children are less susceptible to exercise-induced muscle damage than adults when performing bench press exercise [51], one leg–stepping exercise [52] plyometric exercise [48, 82] and resistance exercise performed with eccentric-type muscle actions [50].

The physiological meaning of increased serum CK following various forms of exercise has been recently reviewed [87] and consensus seems to be that increased levels following exercise may not always be indicative of muscle damage. Furthermore, CK levels cannot be used to ascertain the recovery from a bout of exercise, as it doesn’t necessarily relate to performance measures and it is evidently not highly sensitive in young athletes. In fact, the levels of CK induced by sporting activities [87] may induce myositis but are typically nowhere near the values recorded in case of rhabdomyolysis and up to 300 % lower than the increases observed following myocardial infarct [88] or in burn patients [89].

It has been shown that the activities of the anaerobic enzymes creatine kinase, adenylate kinase, and lactate dehydrogenase (LDH) in children are lower than in adults—possibly linked to the fact that children have a greater ability to oxidize lipids during exercise and have less reliance on anaerobic metabolism [90]. Hence, if CK is used as a marker to ascertain the metabolic implications of training sessions in children and adolescents, it is likely that such an approach might not reflect the true extent of the effects of the training bout on skeletal muscle. The repeated bout effect observed in children and adolescents is also different than in adults. In fact, this occurs more readily to eccentric exercise in adolescent boys than adults [82] which may cloud any measurements made in a crossover design to assess the effectiveness of CWI.

Muscle fiber types should also be considered. Although there is some controversy regarding fiber type differences between children and adults [80, 91–93], there are only minor increases in the proportion of type II fibers with age [80, 91, 92]. Even with a greater proportion of type I muscle fibers in children, no strong correlation between fiber type composition and enzyme activity in skeletal muscle exists [94]. Nonetheless we know that fast-twitch muscle fibers are the ones susceptible to muscle damage and there are a low number of them in adolescent groups [46]. Muscle architecture differences between boys and adults should also be considered as previous work has postulated that a shift to a longer muscle length makes muscle less susceptible to damage [95, 96]. For these reasons, it seems evident that knowledge regarding muscle damage and remodeling in children and adolescents involved in routine, structured, sporting training is somewhat lacking. In particular, when it comes to the need of implementing recovery strategies, it is not clear if interventions like CWI might actually be detrimental to long-term developments.

Adolescent populations display reduced symptoms of muscle damage, perhaps in relation to a reduced nociceptive tolerance (i.e. they cannot work for as hard or as long or endure as high a relative level of pain). The perceived beneficial effects of CWI could also be due to placebo effects and expectation of a positive outcome. Adolescent athletes may experience the ‘pain’ of recovery as a challenge in which the perceived benefits are greater than the demands [97, 98]; the moderate pain of a cold immersion may be reported as pleasant and aid the placebo effect as its outcome is perceived as less painful than doing nothing [99]. Building expectations that pain following training needs recovery interventions, as typically justified by coaches/parents might contribute to the positive effects perceived and prolonged unnecessary exposure can be sought due to peer pressure, which may be counterproductive and defeat the purpose of the intervention. Studies in young individuals have linked pain and guilt and participants with induced feelings of guilt were able to endure submersion of their hand in ice-water for longer compared to those who weren’t guilt ridden [100].

## General physiological effects of cold applications

### Cold-water immersion

**Fig. 1:**
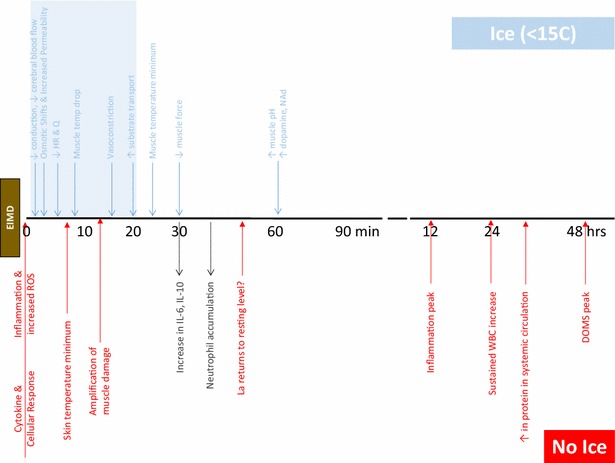
Diagram of effects post exercise with and without icing. *ROS* reactive oxygen species, *HR* heart rate, *Q* cardiac output, *IL-6* interleukin-6, *IL-10* interleukin-10, *WBC* white blood cells, *DOMS* delayed onset muscle soreness

CWI has typically been identified as using water temperatures under 15 °C [101]. The protocols consist of immersing the lower limbs and/or the whole body up to the neck. This temperature may not drastically affect core temperature with the relatively short immersion times used. Studies conducted to ascertain the effects of CWI on hypothermia in humans, refer to periods under 3 min as the initial immersion with short term being up to 15 min and long term greater than 30 [102]. The time course of various physiological processes involved in muscle remodeling post exercise have been extensively reviewed previously [103–107] and we have summarized them with a schematic diagram (see Fig. 1) to highlight how CWI can influence such processes.

During immersion, the major heat exchange occurs via conduction with the surrounding water and hence the level of immersion is important as this dictates the body surface area in contact with the surrounding medium. Within an adult population as core body temperature decreases, so does heart rate [108] causing concomitant decreases in cardiac output [109, 110] and increases in peripheral resistance and arterial blood pressure [111]. In order to preserve core temperature, the body alters blood flow, metabolism and oxygen consumption [109, 112] to make sure homeostasis is maintained.

Localised vasoconstriction reduces fluid diffusion into the interstitial space, which can assist in reducing muscle damage and acute inflammation. Typically the cooler the water, the greater the core-to-water temperature gradient and hence the rate of cooling [113]. It may not be necessary to use very cold-water to reduce blood flow as a comparison of 8 and 22 °C CWI showed a 40 % reduction in both conditions [114]. Skeletal muscle’s blood flow is not the only parameter affected by CWI. In fact, the neuromuscular system is also affected, as tissue cooling decreases the transmission rate along neurons by decreasing the production of acetylcholine [115]. This has been shown to reduce both pain perception [116, 117] and muscle spasm [39, 118]. While this can be of benefit to recovery it must be considered in a practical context as cooling muscles can decrease muscular contractile speed and the force generating ability of the athlete directly after cold immersion [119, 120].

Within an adult cohort core temperature changes are dependent on a number of factors; the method of immersion [121], the duration [122, 123] and the adiposity of the individual involved [124, 125]. Regardless there is a relatively consistent pattern with skin temperature dropping rapidly in the first 3 min and reaching the minimum after 8-9 min [121]. Superficial intramuscular temperature cools faster than deep muscle tissue with the magnitude of change being proportional to the thermal gradient [126]. Given the typical duration of immersion in practical use the deeper tissues will reach a temperature minimum once cooling has actually ceased as heat continues to be lost to the superficial tissues surrounding them even after subjects leave the water [126–128]. This suggests what the athlete does immediately after immersion is important—modest cryotherapy (~20 °C) of a large mass may be successful if there is a period out of the water to allow this temperature ‘afterdrop’ to occur, i.e. the athlete doesn’t immediately leave the cold-water and have a hot shower or bath to ‘re-warm’. This may promote an intermittent or contrast-cooling technique as colder temperatures may be possible whilst limiting the potential for injury to superficial tissues. Alternatively this may promote a strategy of agitation of the immersion medium to prevent a ‘barrier layer’ forming around the subject and promote convective heat loss. Alternatively it may not be an issue in an adolescent population given their reduced adiposity, muscle mass and limb girth in comparison to an adult group. The timing of immersion in relation to exercise may also be important as high intensity exercise can create a greater thermal gradient.

### Skeletal muscle metabolism

Enhanced metabolite clearance and reduced inorganic phosphate and hydrogen ion accumulation has been shown within skeletal muscle following CWI [43]. Reduced muscle pH is a contributing factor to peripheral fatigue development and the subsequent reduction in its force-generating capacity [129]. Cooling skeletal muscles has been shown to result in a reduced acidosis as measured by ^31^P magnetic resonance spectroscopy one hour after exercise [130]. Any benefit of cold-induced pH restoration could be concealed by the reduced functional capacity of a cold muscle [131]. Similarly, but with a different method (hyperoxia) Vanhatalo and colleagues showed that the [PCr] fall during constant work rate knee extension took 50 % longer and the critical power was 10 % higher compared with normoxia—though the terminal values of [PCr] and pH were the same [132]. PCr recovery has been shown to be positively associated with age [133] again giving an indication that at a similar [PCr] children recover quicker than adults.

Few studies have investigated sham or placebo affects relative to CWI—as of course cooling is difficult to blind to. The placebo effect is a well-accepted phenomenon within medicine and also influences sport performance [134], and has a potentially long-lasting effect [135]. Utilising thermoneutral conditions with and without the addition of a placebo and CWI to measure recovery from 4 × 30 s sprints in male adults, Broatch and colleagues found that the placebo condition is as effective as CWI for the recovery of muscle strength despite there being no difference in blood markers [136]. The absence of a difference indicates that perception of recovery with such modalities might have a larger effect than any physiological effect per se.

### Cardiovascular Implications for cold-water immersion

Partial and head-out immersion can alter heart activity [108, 137–139] as well as restore central blood volume and enhance cardiac pre-load—though it has been suggested that a large proportion of the body mass needs exposed for cardiovascular changes to occur [18]. Within an adolescent population it has been shown that CWI may normalize [138, 140] or even enhance parasympathetic heart rate modulation and restore vagal tone following intense exercise similar to the mechanism in an adult population [141]. However, to date, the information on the implications of CWI on adolescent athletes is somewhat limited.

### Endocrine system

Exercise and CWI alter the hormonal milieu of the body and in turn modulate blood flow, fluid balance, heart rate and breathing frequency among other physiological parameters. Marked increases in the circulating levels of dopamine and noradrenaline with no concomitant change in cortisol have been found after immersion for 1 h in 14 °C water. This suggests that prolonged (1 h) head out immersion in such temperature is capable of triggering marked responses in the adrenergic system but limited effects on the hypothalamus-hypophysis axis in young males (22.2 ± 2.4 years), however the physiological significance of such marked acute changes is unknown [108].

## Review and meta-analysis methods

In order to quantify the effectiveness of this recovery modality on various outcomes in young athletic populations, a computerized literature search was conducted, ending in February 2015, using Medline, SportDiscus, and ISI Web of Knowledge. The following key phrases and their combinations were used: cold, immersion, ice, water, recovery, training, adaptation, youth, elite, sport, exercise and child*. Reference lists of all articles were examined for identification of further eligible studies. 259 articles were identified from the initial literature search. Of these 133 were discarded as not being directly relevant to the area. 19 additional articles were identified from the reference lists of appropriate studies.

Priority was given to articles dealing with adolescent athletes (where the average age of the study participants were ≤20 years). This meant there were 17 articles included in this systematic review. We also focused on immediate use of cold-water therapies rather than delayed interactions.

### Assessment of study quality

**Fig. 2:**
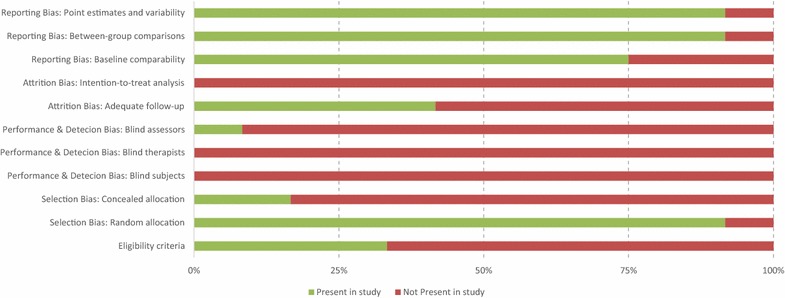
Risk of bias summary as measured via PEDro

Study quality and risk of bias was assessed using the PEDro scale [142]. Any papers not already rated on the PEDro database were scored by the two authors independently and the moderated score was used. The PEDro scale is based on “expert consensus” not, for the most part, on empirical data and papers can be rated on a scale from 1 to 10. Each study was allocated a score based on; eligibility criteria, subjects being randomly allocated to groups, using a concealed allocation, similar baseline groups for the most important prognostic indicators, blinding of all subjects, therapists and assessors, measures of at least one key outcome were obtained from more than 85 % of the subjects initially allocated to groups, all subjects for whom outcome measures were available received the treatment or control condition as allocated, the results of between-group statistical comparisons are reported for at least one key outcome, the study provides both point measures and measures of variability for at least one key outcome. This allowed an assessment of risk of bias (Fig. 2).

### Study inclusion and exclusion criteria

Studies meeting the following criteria were considered for review: (1) The study design was randomised into an intervention group and a control group; (2) only outcome variables measured at 24 h or less or 96 h or greater post exercise were included; (3) outcome measures were taken longitudinally and not simply pre and post a chronic training period or serial recovery interventions; (4) the intervention was applied within 1 h post exercise and permitted to be repeated on subsequent days and (5) participants could be male or female and of any athletic training status. Studies were excluded when (1) the control group received an intervention with rationale to improve recovery; (2) the observed effect could not specifically be attributed to intervention group (e.g., CWI group received multiple interventions, such as active recovery and CWI); (3) there were insufficient data to report an effect size; (4) if the intervention happened pre exercise and (5) if no longitudinal data was reported for recovery.

### Meta-analysis methods

All meta-analyses calculations were conducted using an open source software (Open Meta-Analyst, Center for Evidence-Based Medicine, Brown University School of Public Health, USA). The standardized mean differences were calculated (Hedges *g*) along with 95 % CI using data extracted from the manuscript to compare the intervention group to the control. If these values were only displayed in figures, then a commercially available software (Graph Digitizer v2.26, Datatrend Software, Raleigh, North Carolina, USA) was used to digitize the graphs and extract the means and standard deviations. Effect sizes of <0.2, <0.5, <0.8, >0.8 were considered trivial, small, moderate and large, respectively [143]. The numbers of studies proved too small to differentiate findings between pre and post pubertal populations so the analysis was performed for all studies with participants under 20 years of age. As a common effect size cannot be assumed where we are combining the data under broader headings, the random-effects model for the meta-analysis of pooled data was implemented. Heterogeneity was assessed using the I^2^ statistic, which describes the percentage of variability in effect estimates that is due to heterogeneity rather than chance. I^2^ values of 25, 50 and 75 % respectively represent low, moderate and high statistical heterogeneity.

The outcome variables were grouped by type according to the observed effects. Within the ‘Physiological’ group we included measures of tympanic temperature, heart rate (HR), lactate (La), bicarbonate (HCO_3_), creatine kinase (CK) and lactate dehydrogenase (LDH) where appropriate. We utilized fatigue, delayed onset muscle soreness (DOMS) and total quality recovery scores (TQR) scores in the ‘subjective’ category and then clustered performance measures of ‘power’ and ‘endurance’ (vertical jump (VJ), squat jump (SJ), countermovement jump (CMJ), peak power (PP), speed, agility & maximal voluntary contraction (MVC) and repeated sprints, time trials, total distance and shuttle run scores respectively). We then categorized these as immediate or delayed effects (immediate being <24 h and delayed >96 h).

## Results of the meta-analysis: overall effects of water immersion on recovery in adolescent athletes

The published literature within an adolescent population encompasses 19 studies based on age criteria alone. One study was discounted as it utilized multiple interventions (combined sauna, CWI and jacuzzi) for recovery [144]. One was discounted as the control group was active to promote recovery [145]. Two were discounted as they utilized pre-cooling rather than cooling as a recovery intervention [146, 147], one for the absence of a control group [148] and two as they utilized chronic recovery interventions [149, 150]. This left 12 studies included in the review. Of those remaining, only 10 studies utilised CWI (Additional file 1: Table S1); [140, 151–158] and 6 studies utilised protocols alternating hot and cold-water immersion techniques [149, 151, 155, 159–161]. Of these studies five looked at the intervention versus a passive control [128, 140, 152–154], 3 against a thermoneutral immersion control [156–158] and the remaining four against each other and a passive control [151, 155, 159, 160]. Within these studies the outcome variables were diverse as well as the research design. Passive intervention was defined as either seated rest or no intervention. Across studies the main differences came in temperature, immersion depth and total time (Additional file 1: Table S1). The CWI ranged from 8 to 15 °C in all of the studies (the majority being 10–15 °C with one under 10 °C). The total duration of immersion ranged from 5 to 15 min with a median value of 10, (mean = 10.14 ± 2.70 min). Five of the studies utilized a single immersion [128, 140, 151, 154, 158] with the remainder using repeated periods of immersion to reach the total time. In all of the 12 studies assessed we can consider the immersion periods to be short term (i.e. <15 min). The depth of immersion, when stated, ranged from the fibular head to the shoulders though most commonly this was somewhere within the thorax region with complete immersion of the lower limbs.

**Fig. 3:**
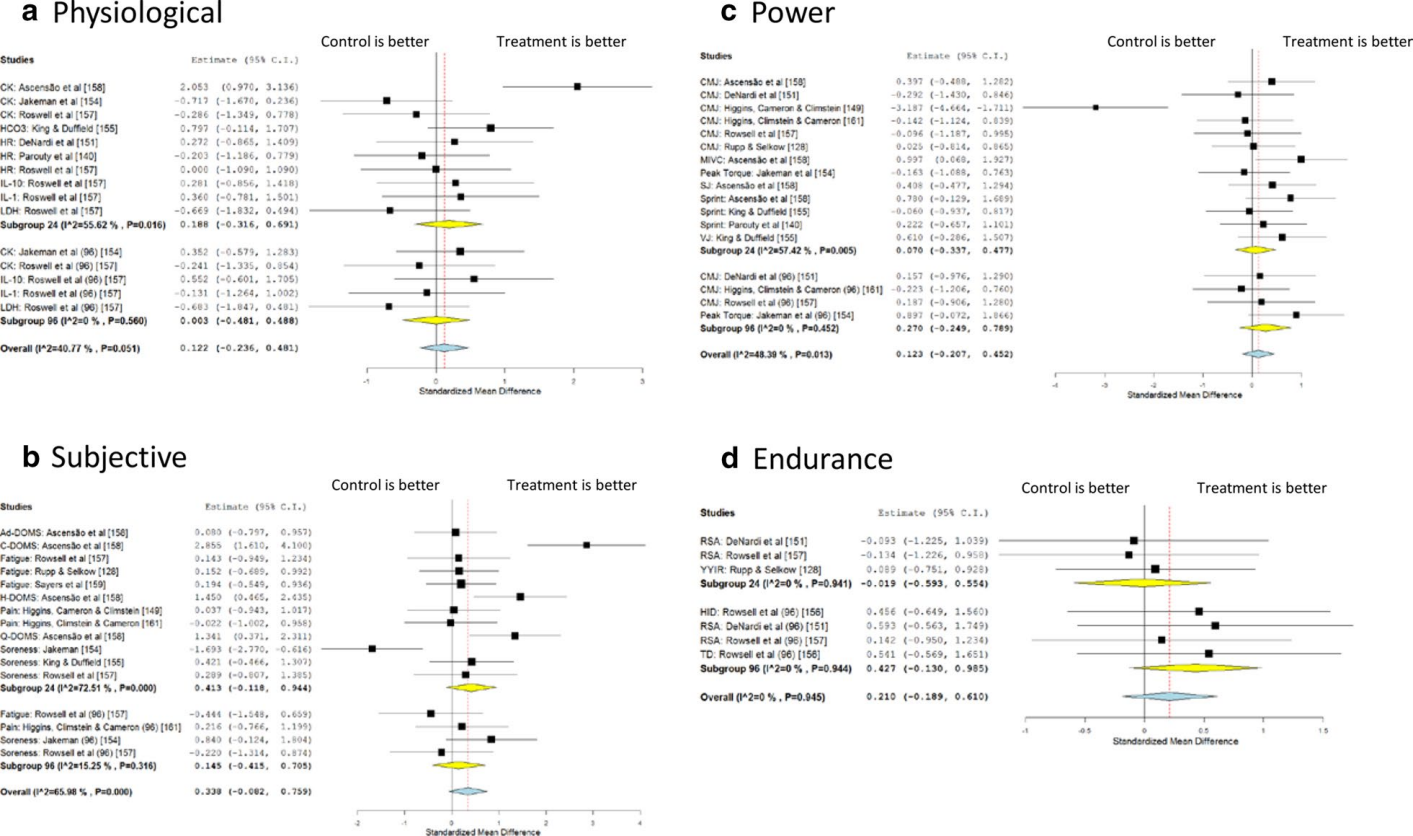
Meta analysis of effects of cold water immersion on different areas. *CK* creatine kinase, *HCO3* bicarbonate, *HR* heart rate, *IL-10* interleukin-10, *IL-1* interleukin-1, *LDH* lactate dehydrogenase, *CMJ* counter movement jump, *MVC* maximal voluntary contraction, *SJ* squat jump, *VJ* vertical jump, *RSA* repeat sprint ability, *YYIR* Yo–Yo intermittent recovery, *HID* high intensity distance, *TD* total distance, *Ad-DOMS* adductor delayed onset muscle soreness, *C-DOMS* calf delayed onset muscle soreness, *H-DOMS* hamstring delayed onset muscle soreness, *Q-DOMS* quadriceps delayed onset muscle soreness

The meta-analysis of the published literature would suggest that overall effect sizes with CWI are trivial to small. Looking at acute outcomes, the only evident benefit appears to be in the subjective outcome measures (Fig. 3b, ES = 0.41, 95 % CI −0.12, 0.94) suggesting that perception of its beneficial effects seems to be the predominant outcome in particular when measured with perception of soreness scales and/or DOMS perception scales. For other areas, the effect sizes are negligible; Physiological (Fig. 3a, ES = 0.19, 95 % CI −0.32, 0.69) Power (Fig. 3c, ES = 0.07, 95 % CI −0.34, 0.48), Endurance (Fig. 3d, ES = -0.02, 95 % CI −0.59, 0.55) outcome measures do not seem to provide strong evidence to the use of CWI as a recovery modality in young athletes. In particular, in physiological measures, CK seems to be the marker mostly affected by CWI as directly linked to DOMS and perception of pain, with other outcome measures being less affected by this treatment. In our cluster of power outcome measures, CMJ and sprinting ability seem to have a superior ability to be affected by CWI interventions as compared to isometric and/or isokinetic outcome measures. The endurance outcome measures present problems with their large variability and therefore, considering the likelihood of small effects of both interventions may not be sensitive enough to detect meaningful changes. Based on our analysis, the current literature therefore suggests no significant effects of CWI in restoring and/or maintaining performance in a young population.

In terms of delayed effects, again there is limited support for using CWI in this population, though there may be a small effect on endurance outcomes (ES = 0.43, 95 % CI −0.13, 0.99) however the evidence comes from only 3 studies.

**Fig. 4:**
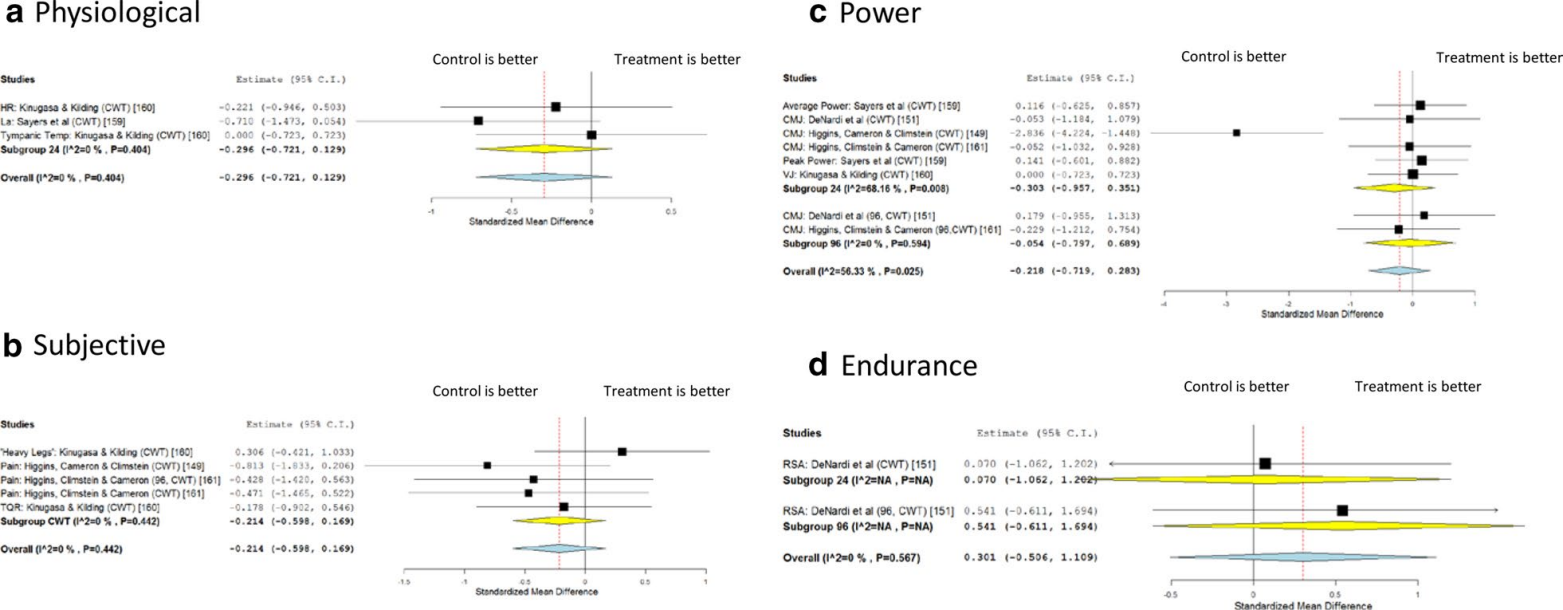
Meta analysis of effects of contrast water therapy on different areas. *HR* heart rate, *La* lactate, *TQR* total quality recovery, *CMJ* counter movement jump, *VJ* vertical jump, *RSA* repeat sprint ability

For contrast therapies a similar negative pattern emerges—in the Physiological area there is a small detrimental effect acutely (Fig. 4a, ES = −0.30, 95 % CI −0.72, 0.13). There is only 1 study looking at longer term subjective effects which also shows a small negative effect (Fig. 4b). The opposite is true of the one study looking at Endurance markers with a moderately positive effect (Fig. 4d, ES = 0.54, 95 % CI −0.61, 1.69). The acute effects on power are negative and small (Fig. 4c, ES = −0.30, 95 % CI −0.96, 0.35). The literature therefore suggests no significant benefits of CWT in restoring acute performance.

### Quality of the evidence

The PEDro ratings indicated an average score of 4.17. This would suggest that the quality of the trials so far published is low to moderate. There appears a case for selection bias as only 2 studies utilized a concealed allocation suggesting that this is an area that can be addressed to improve the quality of studies in the area (Fig. 2). One of the main issues to be solved before reaching consensus in this area is the limitations and quality of the studies so far conducted. While this is easy to criticize, it is important to remind the readers that the design of proper randomized controlled studies is made challenging by the fact that individuals cannot be blind to the cold water intervention, however multiple interventions involving water immersion of different temperature could be used and for sure the scientists performing the measurements could and should be blind to the treatments when assessing its effects in order to reduce bias and improve the quality of the results presented. Finally, considering the relatively small sample sizes of the studies and the small effect sizes identified in this meta-analysis, it seems clear that larger scale studies are needed.

### Short and long-term adaptation

Recent reviews have concluded that the literature suggests CWI may attenuate adaptations to training; however, due to the methodology employed further research investigating the effects on adaptation for athletic performance is required [19]. Recent work in healthy young adults performing strength training programmes suggests that CWI can attenuate adaptations to training in comparison to active recovery over 12 weeks [162] and in comparison to simply not cooling over 6 weeks [163]. These preliminary studies as well as previous work [164] indicate that rapid reduction in temperature with a consequent reduction in swelling and inflammation of the affected areas could impair the improvement in skeletal muscle form and function following resistance exercise programmes.

Therefore a cautious approach to the use of interventions aimed at reducing the exercise-induced inflammatory responses is needed and should be based on the desired outcome and the health, age and training status of the individual. In the case of young athletes, manipulations of training loads and exercise modalities should be sought to make sure that the progressions and loading patterns are adequate to the age and training experience and recovery strategies should not need to be employed to guarantee excessive loads.

It is unknown if CWI can be detrimental for adolescent athletes trying to increase strength and muscle hypertrophy as at the moment there is no study which has addressed this aspect in this population. We do not know much about the long-term effects of repeated exposures to CWI and so caution should be exercised when repeatedly using this modality in an adolescent population. While the use of this recovery modality may allow a faster recovery rate and also facilitate increased training volume and intensity one wonders if the same level of adaptation may occur without the use of CWI. For sure practitioners should always ask themselves if the intervention is appropriate for the training outcomes and if the risks outweigh the benefits.

### Recommendations

Based on the findings from this review and meta-analysis it would appear that the effects of CWI on young athletes are small to non-existent either acutely or over a few days after the exercise bout (i.e. >96 h). The current literature provides only a small number of studies characterizing the acute and chronic responses to CWI and CWT in adolescent athletes (221 subjects in total across each condition) and as a consequence it is difficult to draw definite conclusions. While most protocols currently implemented in the field are based on findings on adult populations and suggest positive effect in reducing DOMS [21], no hindering of glycogen re-synthesis [129] and no attenuation in endurance capacity when applied during a training period [165] currently the knowledge is too limited regarding adolescent athletes with reference to muscle remodeling as a consequence of training.

It is clear that there needs to be a consensus reached on the temperature and methodologies employed and indeed there would appear to be a need for characterization of an individual’s response to both a training session and the use of recovery interventions. Cold itself is not likely to affect recovery from all types of exercise uniformly. CWI protocols of excessive duration may actually exacerbate the concentration of cytokines in circulation post-exercise [18]. It may be that similar to hormesis there is a low-dose stimulation, high dose inhibition relationship.

This highlights the need to differentiate the use of CWI within training and/or competition, which is further complicated by the influence of growth and maturation in the adolescent athlete. It may be that due to their lower relative power young athletes cannot create enough ‘stress’, to need to recover from. Furthermore, preventing inflammation may actually hinder recovery [166] so in some cases it may be better to avoid using interventions to reduce the inflammatory responses to allow adaptation to training and consider CWI applications only when a high density of competitive events (i.e. competition phases), inducing DOMS are planned as such an approach might promote faster recovery.

Future studies should utilize modern non-invasive technologies to characterize current practices of coaches in relation to adolescent athletes. The use of minimally and non-invasive technologies could overcome the ethical issues around invasive methods in this population and provide practical tools to ascertain the efficacy and the need of CWI. Techniques like thermal imaging to detect blood flow and track the development of DOMS [167], tensiomyography and twitch interpolation techniques to assess evoked responses and salivary assays might provide a solution to understand more about the appropriateness of such recovery methods with a psychobiological approach as it seems clear that perception of recovery might be affected.

## Conclusions

Recovery activities should be part of a well-planned, controlled and managed training programme. This is important not only for elite senior athletes but it is of particular relevance for young athletes involved in full time sports academies and/or engaged in structured long term development plans. The popularization of practices by the media means that protocols and procedures in place for elite senior athletes can be extended to adolescent performers without an appropriate knowledge of the risk and reward. CWI is a popular intervention used for “recovery” by many athletes/coaches. Asking the question “recovery from what?” before thinking about its use should be the first step before deciding if it is appropriate to use it. However, when young athletes (in particular pre- and peri-pubertal) are to be exposed to such practice, it is important to understand the time and the place for the use of CWI and most of all be aware of the current knowledge (or lack of it) about the implications for using such interventions. Recovery is a holistic process which should consider the acute implications of recovery strategies in the context of the long term outcomes of a training programme. So, when planning the use of recovery modalities, practitioners, coaches and parents should understand that physical training is characterized by stressors which stimulate our bodies to adapt. In the case of skeletal muscle, producing the final result of stronger and better functioning muscles. When intervening to accelerate such processes and/or amplify the potential for adaptation, we need to make sure that the intervention used is safe, effective, and it does not harm the long term potential for adaptation in a young athlete. According to our literature review and meta-analysis, more work is needed to ascertain the impact and effectiveness of cold applications on a pre or peri-pubertal population, so until more and better evidence becomes available the advice to young athletes can be the same that our ancestors suggested: “Perfer et obdura, dolor hic tibi proderit olim” (*“*Be patient and tough; someday this pain will be useful to you*”*. Ovid, Amores, Book III, Elegy XI).

## Additional file

10.1186/s13728-015-0035-8  Summary from included adolescent studies.

## Abbreviations

CK: creatine kinase
CMJ: countermovement jump
CWI: cold-water immersion
CWT: contrast water therapy
DOMS: delayed onset muscle soreness
EIMD: exercise induced muscle damage
HCO_3_: bicarbonate
HR: heart rate
IL-1: interleukin-1
IL-10: Interleukin-10
IL-6: interleukin-6
La: Lactate
LDH: lactate dehydrogenase
MVC: maximal voluntary contraction
PEDro: physiotherapy evidence database
PP: peak power
ROS: reactive oxygen species
SJ: squat jump
TQR: total quality recovery
VJ: vertical jump

## Footnotes

1 Defined as age 10 to 19 by the United Nations (http://www.un.org/esa/socdev/documents/youth/fact-sheets/youth-definition.pdf).
