# Supplementary figures and images for: Cold applications for recovery in adolescent athletes: a systematic review and meta analysis
Source: Extrem Physiol Med. 2015 Oct 12;4:17. doi: 10.1186/s13728-015-0035-8 (PMC4603811; doi:10.1186/s13728-015-0035-8)

## Slide 1
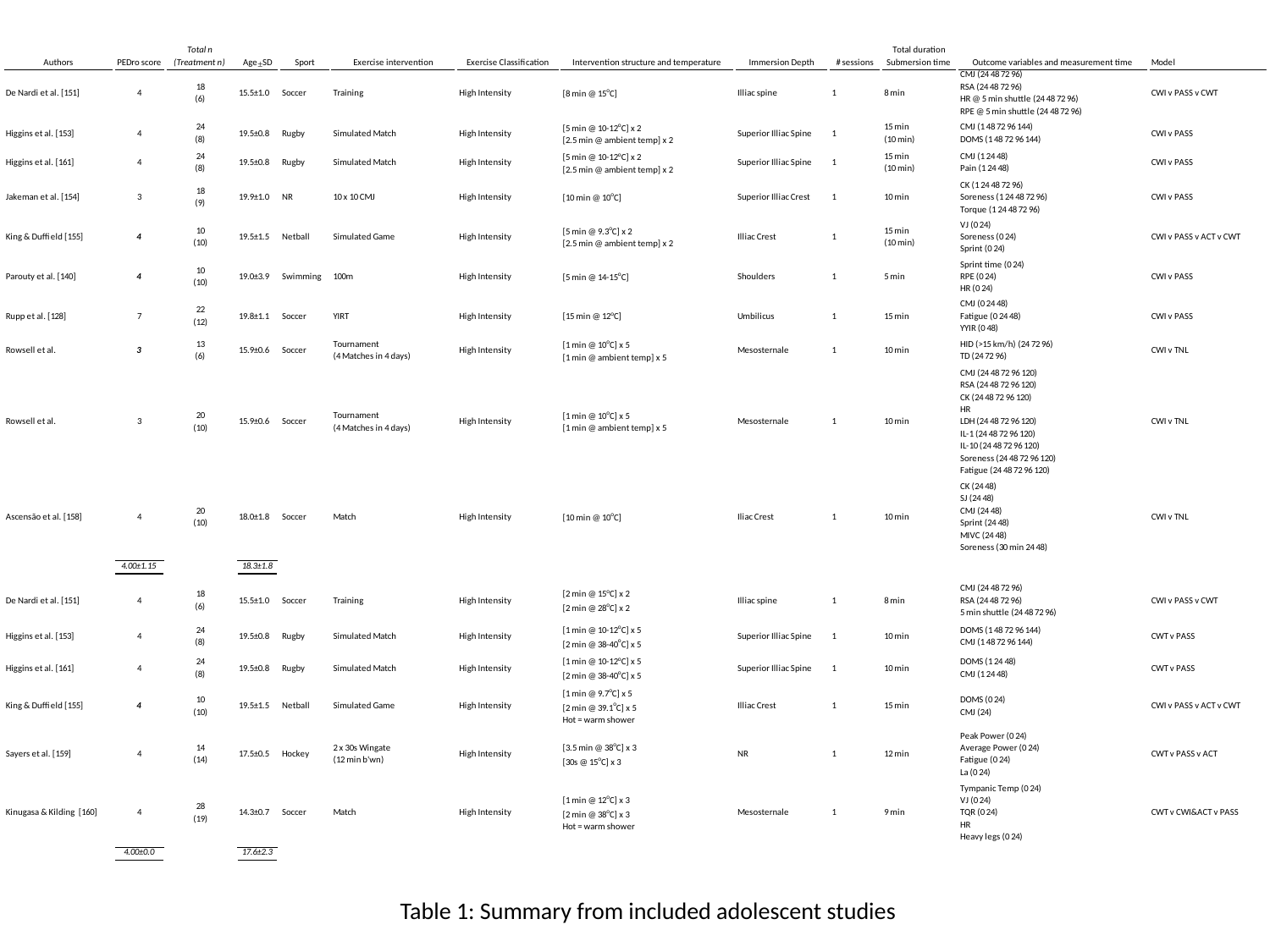

Table 1: Summary from included adolescent studies

Supplement: Supplementary file 1 — 10.1186/s13728-015-0035-8 Summary from included adolescent studies. [file 13728_2015_35_MOESM1_ESM.pptx]
